# Characterization of Egg Laying Hen and Broiler Fecal Microbiota in Poultry Farms in Croatia, Czech Republic, Hungary and Slovenia

**DOI:** 10.1371/journal.pone.0110076

**Published:** 2014-10-16

**Authors:** Petra Videnska, Md. Masudur Rahman, Marcela Faldynova, Vladimir Babak, Marta Elsheimer Matulova, Estella Prukner-Radovcic, Ivan Krizek, Sonja Smole-Mozina, Jasna Kovac, Ama Szmolka, Bela Nagy, Karel Sedlar, Darina Cejkova, Ivan Rychlik

**Affiliations:** 1 Veterinary Research Institute, Brno, Czech Republic; 2 Faculty of Veterinary Medicine, University of Zagreb, Zagreb, Croatia; 3 Biotechnical Faculty, University of Ljubljana, Ljubljana, Slovenia; 4 Institute for Veterinary Medical Research, Hungarian Academy of Sciences, Budapest, Hungary; 5 Department of Biomedical Engineering, Brno University of Technology, Brno, Czech Republic; Catalan Institute for Water Research (ICRA), Spain

## Abstract

Poultry meat is the most common protein source of animal origin for humans. However, intensive breeding of animals in confined spaces has led to poultry colonisation by microbiota with a zoonotic potential or encoding antibiotic resistances. In this study we were therefore interested in the prevalence of selected antibiotic resistance genes and microbiota composition in feces of egg laying hens and broilers originating from 4 different Central European countries determined by real-time PCR and 16S rRNA gene pyrosequencing, respectively. *strA* gene was present in 1 out of 10,000 bacteria. The prevalence of *sul1*, *sul2* and *tet(B)* in poultry microbiota was approx. 6 times lower than that of the *strA* gene. *tet(A)* and *cat* were the least prevalent being present in around 3 out of 10,000,000 bacteria forming fecal microbiome. The core chicken fecal microbiota was formed by 26 different families. Rather unexpectedly, representatives of *Desulfovibrionaceae* and *Campylobacteraceae*, both capable of hydrogen utilisation in complex microbial communities, belonged among core microbiota families. Understanding the roles of individual population members in the total metabolism of the complex community may allow for interventions which might result in the replacement of *Campylobacteraceae* with *Desulfovibrionaceae* and a reduction of *Campylobacter* colonisation in broilers, carcasses, and consequently poultry meat products.

## Introduction

Poultry meat is the most common protein source of animal origin for humans. However, extensive breeding and selection for increased meat or egg production together with animals living in confined spaces have led to an increased susceptibility of chickens to infections with pathogens, some of them with zoonotic potential. Moreover, if an infected flock is treated with antibiotics, then the antibiotic resistant bacteria are positively selected and the poultry products become contaminated with such bacterial clones.

The composition of gut microbiota is known to affect many host functions including nutrient utilization, gut epithelium nourishment and the development and activity of the gut immune system [1]–[4]. One of the possibilities how to reduce pathogen colonization as well as antibiotic usage in poultry production is therefore to maintain a normal gut microbiota composition. This can be achieved either by using an appropriate feed formula or by providing chickens with live beneficial bacterial cultures. However, most of the experiments in this area were based on an empirical basis as it is quite difficult to determine the effect of any such preparations on gut microbiota composition when culture conditions for the majority of gut microbiota are not known.

Since the introduction of next generation sequencing, culture independent characterization of gut microbiota has become possible and has allowed for the characterization of complex microbial communities including those in the intestinal tract of chickens. We and others have already characterized changes in chicken gut microbiota after antibiotic therapy, pathogen infection or throughout the chicken's life [5]–[8]. Besides understanding the behavior of gut microbiota under extreme conditions such as the administration of antibiotics, it is also important to understand what is normal and common to at least the majority of poultry flocks. Accumulation of such knowledge will then allow for a gradual definition of an aberrant microbiota composition and setting up of experiments towards the selection of microbiota with a positive effect on host performance.

In this study we therefore tested to what extent egg or meat production systems in different Central European countries may affect the composition of chicken fecal microbiota and the prevalence of antibiotic resistance genes in whole bacterial population. We considered 2 alternative hypotheses. First, that the microbiota composition would be highly diverse among the countries in which case we would continue with a detailed epidemiological investigation. The alternative hypothesis was that the microbiota composition would not be extremely different, under which circumstance we should be able to define core chicken microbiome. To test both hypotheses we collected over 100 fecal samples from both broilers and egg layers in 4 different countries in which we compared the prevalence of antibiotic resistance genes as well as the composition of fecal microbiota. As the fecal microbiota composition of broilers and hens across four EU countries was quite similar, we finally defined bacterial families representing core chicken fecal microbiome.

## Results

### Antibiotic resistance gene prevalence in fecal microbiota of egg layers and broilers

**Figure 1:**
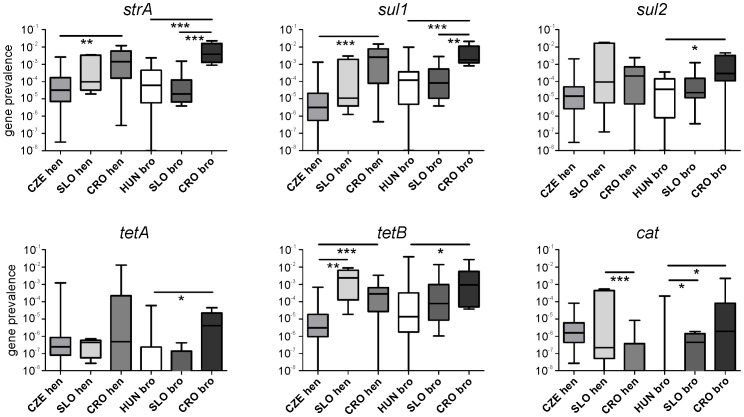
Antibiotic resistance gene prevalence in poultry fecal microbiota. Antibiotic resistance gene prevalence is presented as the median with 25^th^ and 75^th^ percentile (box) and the whiskers indicating minimum and maximum values recorded. Mind the logarithmic scaling of Y axis.

The *strA* was the most prevalent gene in all the samples. The median prevalence of this gene in poultry microbiota was around 0.0001 which means that it was present in 1 copy per 10,000 copies of 16S rRNA genes. Since copies of 16S rRNA genes approximately correlate with a number of bacteria, *strA* gene was present in 1 out of 10,000 bacteria forming fecal microbial population. The prevalence of *sul1*, *sul2* and *tet(B)* in poultry microbiota was approx. 6 times lower than that of the *strA* gene and *tet(A)* and *cat* genes were the least prevalent, being present in around 3 out of 10,000,000 bacteria. When the samples were compared according to country of origin, those of Czech or Hungarian origin usually had the lowest antibiotic resistance gene prevalence. On the other hand, *strA* and *sul1* genes were significantly increased in broiler and egg layer microbiota from Croatia whilst an increased prevalence of *tet(B)* or *cat* was characteristic of the microbiota of egg laying hens from Slovenia. When the microbiota of broilers was compared with the microbiota of egg layers, no significant differences were observed in the prevalence of antibiotic resistance genes (Fig. 1 and Table S1).

### Microbiota composition determined by 16S rRNA pyrosequencing

**Figure 2:**
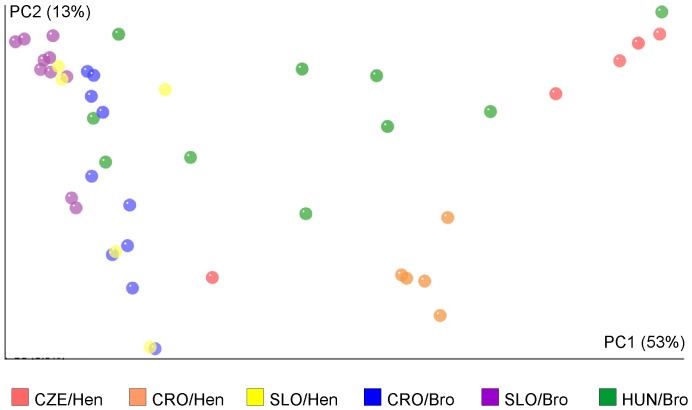
Microbiota diversity in fecal samples of broilers and hens. UniFrac analysis followed by PCoA showed that the two main factors explained 69% of the variability observed in the microbiota composition in poultry fecal samples. Slovenian egg layer microbiota were similar to samples originating from broilers and Hungarian samples of broiler origin clustered between broiler samples from Slovenia and Croatia, and egg laying hen samples from Croatia and the Czech Republic. SLO – Slovenia, CRO – Croatia, CZE – Czech Republic, HUN – Hungary, Hen - samples from egg laying hens, Bro – samples from broilers.

Because of the differences in the antibiotic resistance gene prevalence, next we were interested whether these differences were reflected also in microbiota composition. Pyrosequencing of 16S rRNA amplification products in 45 selected samples showed that the microbiota of egg layers was usually more complex than the microbiota of broilers (Table S2), except for the microbiota of hens from Slovenia which was of low complexity, similar to that of broilers (Fig. 2). In both egg layers and broilers, *Firmicutes*, *Bacteroidetes* and *Proteobacteria* represented the major phyla in fecal samples. Broiler microbiota was dominated by *Firmicutes* (76.2%) followed by *Proteobacteria* (14%). *Bacteroidetes* formed only 6.5% of the total fecal microbiota of broilers and *Actinobacteria* were present at 3.8% in broiler microbiota. The microbiota of egg layers was also dominated by *Firmicutes* (58.8%). However, representatives of *Bacteroidetes* formed 22.1% of the total microbiota and *Proteobacteria* formed 16.9%. *Actinobacteria* were present at 0.6% and, instead, *Fusobacteria* were detected as a minority subpopulation reaching 1.4% in egg layer fecal microbiota (for more details see Table S3).

Search for the most common OTUs (Operational Taxonomic Unit, bacterial species with at least 97% similarity in 16S rRNA sequences) showed that 3 OTUs, all belonging to the genus *Lactobacillus* were found in all 45 analysed samples. In addition, other 2 OTUs detected in 43 and 41 samples, respectively, also belonged to the genus *Lactobacillus*. The remaining common OTUs included an unclassified representative of *Peptostreptococcaceae*, and those of genera *Streptococcus* and *Escherichia* (Table 1). However, the more appropriate identification of core chicken microbiota was achieved after a combined view at family and class taxonomic levels. This showed that in addition to the above mentioned genera, representatives of families *Clostridiaceae*, *Ruminococcaceae*, *Lachnospiraceae*, *Veillonellaceae* and classes *Bacteroidia* and *Actinobacteria* were common to chicken microbiota (Table 1).

**Table 1:** List of OTUs and their taxonomic classification found in at least 40 out of 45 samples tested by pyrosequencing 16S rRNA genes.

OTU	Genus	Family	Class	Phylum
***Lactobacillus (45)***	***Lactobacillus (45)***	***Lactobacillaceae (45)***	***Clostridia (45)***	***Firmicutes (45)***
***Lactobacillus (45)***	*Streptococcus (42)*	*Peptostreptococcaceae (44)*	***Bacilli (45)***	***Bacteroidetes (45)***
***Lactobacillus (45)***	*Escherichia (41)*	*Enterobacteriaceae (44)*	***Bacteroidia (45)***	*Proteobacteria (44)*
*Lactobacillus (43)*		*Clostridiaceae (43)*	*Gammaproteobacteria (44)*	*Actinobacteria (42)*
*Peptostreptococcaceae (42)*		*Streptococcaceae (42)*	*Actinobacteria (42)*	
*Streptococcus (41)*		*Ruminococcaceae (42)*		
*Lactobacillus (41)*		*Lachnospiraceae (40)*		
*Escherichia (40)*		*Veillonellaceae (40)*		

In the next step we analyzed whether there are any OTUs which would occur only among hen microbiota and were absent in broiler microbiota, and vice versa. Not a single OTU completely specific for either of the poultry categories was identified. However, the OTUs with the highest preference to either broiler or hen microbiota are listed in Table 2. Data in this table show that OTUs specific to young broilers usually belonged to the phylum *Firmicutes* while those characteristic of adult hens commonly belonged to the phylum *Bacteroidetes*.

**Table 2:** List of OTUs present 10 or more times in either hen or broiler fecal microbiota.

	Reads		Classification			
OTU	Hens*	Broilers*	Phylum	Order	Family	Genus
784	11	0	*Firmicutes*	*Clostridiales*	*Veillonellaceae*	*Megasphaera*
3113	11	0	*Bacteroidetes*	*Bacteroidales*		
4272	11	0	*Bacteroidetes*	*Bacteroidales*		
5509	11	0	*Bacteroidetes*	*Bacteroidales*	*Porphyromonadaceae*	*Parabacteroides*
5896	11	0	*Firmicutes*	*Coriobacteriales*	*Coriobacteriaceae*	*Atopobium*
519	11	1	*Bacteroidetes*	*Bacteroidales*	*Bacteroidaceae*	*Bacteroides*
776	10	0	*Firmicutes*	*Lactobacillales*	*Lactobacillaceae*	*Lactobacillus*
1210	10	0	*Bacteroidetes*	*Bacteroidales*	*Prevotellaceae*	*Prevotella*
1622	10	0	*Bacteroidetes*	*Bacteroidales*	*Bacteroidaceae*	*Bacteroides*
2571	10	0	*Bacteroidetes*	*Bacteroidales*		
4806	10	0	*Bacteroidetes*	*Bacteroidales*	*Paraprevotellaceae*	
7750	10	0	*Firmicutes*	*Clostridiales*	*Veillonellaceae*	*Megamonas*
9129	10	0	*Bacteroidetes*	*Bacteroidales*	*Bacteroidaceae*	*Bacteroides*
3822	10	1	*Bacteroidetes*	*Bacteroidales*	*Bacteroidaceae*	*Bacteroides*
8364	10	1	*Bacteroidetes*	*Bacteroidales*	*Paraprevotellaceae*	*Prevotella*
8059	1	18	*Firmicutes*	*Lactobacillales*	*Lactobacillaceae*	
3467	0	15	*Bacteroidetes*	*Bacteroidales*	*Bacteroidaceae*	*Bacteroides*
2592	0	14	*Bacteroidetes*	*Bacteroidales*	*Rikenellaceae*	
2652	0	13	*Actinobacteria*	*Actinomycetales*	*Corynebacteriaceae*	*Corynebacterium*
4021	1	13	*Actinobacteria*	*Actinomycetales*	*Brevibacteriaceae*	*Brevibacterium*
6880	1	13	*Firmicutes*	*Lactobacillales*	*Lactobacillaceae*	*Lactobacillus*
1226	1	12	*Firmicutes*	*Lactobacillales*	*Lactobacillaceae*	
8226	1	12	*Actinobacteria*	*Actinomycetales*	*Corynebacteriaceae*	*Corynebacterium*
962	0	11	*Firmicutes*	*Lactobacillales*	*Lactobacillaceae*	*Lactobacillus*
2087	1	11	*Bacteroidetes*	*Bacteroidales*	*Rikenellaceae*	
20	0	10	*Firmicutes*	*Clostridiales*	*Ruminococcaceae*	*Ruminococcus*
1373	0	10	*Firmicutes*	*Clostridiales*	*Ruminococcaceae*	
7469	0	10	*Firmicutes*	*Bacillales*	*Staphylococcaceae*	

* numbers show the number of samples in which particular OTU was recorded, out of 15 hen and 30 broiler fecal microbiota samples.

**Figure 3:**
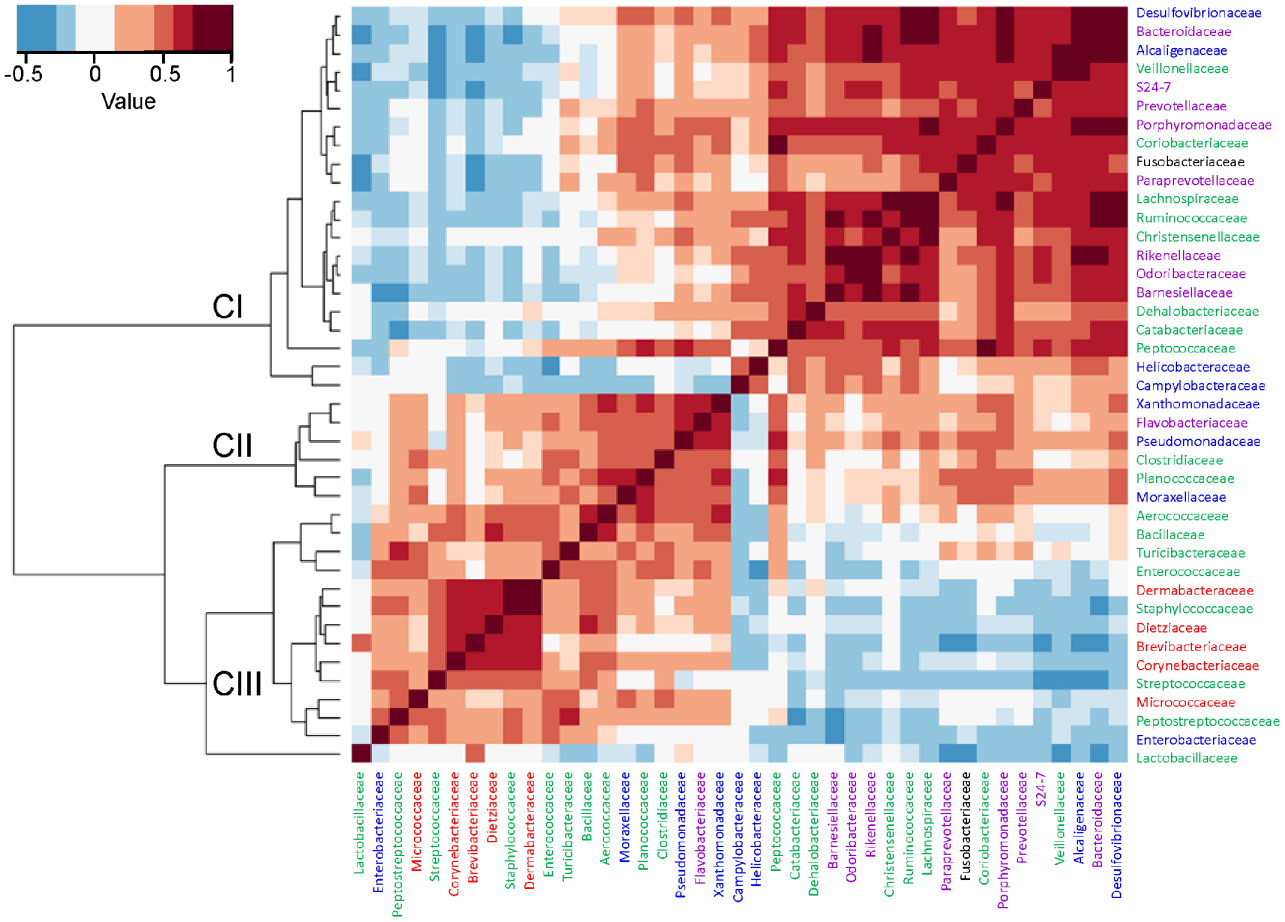
Identification of core chicken fecal microbiota. Cluster I (CI) of positively correlating bacterial families represents families common to established chicken fecal microbiota of adult hens. Interestingly, representatives of *Helicobacteraceae* and *Campylobacteraceae* were clustered with these families, although as the most distant members. Cluster II (CII) was formed mainly by the families which are characteristic of aquatic environments. Cluster III (CIII) of positively correlating bacterial families included bacterial families that were mainly characteristic of gut microbiota of young broilers. Families in green belong to the phylum *Firmicutes*, purple to *Bacteroidetes*, blue to *Proteobacteria* and red to *Actinobacteria*. Shades of brown – positively correlating families; shades of blue – negatively correlating families.

To further identify mutual interconnections among chicken fecal microbiota, the presence of bacterial families was correlated with each other based on the microbiota composition of 45 samples. Although altogether representatives of 108 families were identified in this study in at least one sample, in correlation analysis we included only the families which were present in more than 15 out of 45 analyzed samples, *i.e.* we excluded bacterial families, whose representatives were recorded only sporadically as their inclusion resulted in distorted outputs. This analysis grouped bacterial families into three main clusters (Fig. 3). Cluster I included 21 different families, with equal representation of phyla *Bacteroidetes* and *Firmicutes,* each having 8 families in this cluster. Some of the families, *e.g. Lachnospiraceae*, *Ruminococcaceae*, *Bacteroidaceae*, *Rikenellaceae*, *Veilonellaceae* and *Prevotellaceae*, were frequently reported to be present in the intestinal microbiota of chickens or hens [9]–[11]. The families of Cluster I are therefore characteristic of developed and established microbiota of adult hens. Interestingly, representatives of *Helicobacteraceae* and *Campylobacteraceae* also clustered with these families, although as the most distant members.

Cluster II included 6 bacterial families such as *Pseudomonadaceae*, *Xanthomonadaceae*, *Moraxellaceae* or *Flavobacteriaceae* which we recently detected in the water used for transporting ornamental fish [12] and can be considered as of environmental origin.

The last cluster III included 14 bacterial families, eight of them belonging to the phylum *Firmicutes*, five of them to the phylum *Actinobacteria* and the last one to the phylum *Proteobacteria*. The phylum *Bacteroidetes* was not represented in this group. Families such as *Lactobacillaceae*, *Turicibacteraceaea*, *Corynebacteriaceae*, *Brevibacteriaceae*, *Staphylococcaceae*, *Streptococcaceae*, *Peptostreptococcaceae*, *Enterococcaceae* or *Enterobacteriaceae* were also included in this cluster. These families overlapped with those covering the most common microbiota members listed in Table 1 or those covering microbiota characteristic of broilers (Table 2).

## Discussion

In this study we first analyzed the prevalence of selected antibiotic resistance genes followed by the composition of fecal microbiota in broilers and egg laying hens. The prevalence of antibiotic resistance genes was quite low, similar to our previous report [13]. These results are consistent with the absence of recent therapy in the flocks since the frequencies in antibiotic resistance genes increase during such therapies but decrease very quickly after antibiotic withdrawal [13]. The frequencies of antibiotic resistance genes in different microbial populations vary around similar prevalencies such as 10^−3^–10^−4^, with *sul1*, *sul2* and *strB* genes being usually more frequent than *tet(A)* or *tet(B)* genes [14]–[16]. However, a high prevalence can occasionally be detected as was the case of *sul1* and *sul2* gene prevalence in a report from China reaching prevalence of 10^−2^ in porcine manure and even around 10^−1^ in the manure of chicken origin [14].

Microbiota composition was characterized by pyrosequencing the V3 and V4 variable region of 16S rRNA genes. Though the results can be affected by the efficiency of cell lysis of different bacterial species, efficiency of primer annealing and extension as well as the different rRNA gene copy number in different species [17], [18], pyrosequencing 16S rRNA genes is currently the most frequently used tool for the characterization of complex microbial communities. The microbiota of hens originating from Slovenia differed from the microbiota of hens from the Czech Republic and Croatia (Fig. 2 and Figs. S1 and S2). Although we are unable to explain this observation exactly, we noticed that hens from the Czech Republic and Croatia analyzed in this study were 30 weeks old whilst hens from Slovenia were 61 weeks old. Interestingly, in our previous study, the feces of young hens were quite complex whilst fecal microbiota from older hens consisted mainly of *Lactobacillaceae* [8]. However, in another study where we followed the development of cecal, *i.e.* not fecal, microbiota in hens over their whole life, no such reduction in microbiota composition in the cecum was recorded (unpublished observations). It is possible that the frequency of cecal excretion decreases with increasing age which results in a lower presence of cecal microbiota in the feces. And since *Lactobacillaceae* are present in the small intestine in large quantities [8], [19], these may become enriched also in the feces [20].

In an attempt to identify the core microbiota, all 21 families forming cluster I and selected families from cluster III, namely *Lactobacillaceae*, *Peptostreptococcaceae*, *Streptococcaceae*, *Clostridiaceae* and *Enterobacteriaceae* have to be considered. Although it is likely that additional studies may increase or decrease the number of families defined as the core chicken microbiome, families such as *Lachnospiraceae*, *Ruminococcaceae*, *Clostridiaceae*, *Lactobacillaceae*, *Bacteroidaceae*, *Rickenellaceae*, *Veilonellaceae*, *Prevotellaceae* or *Enterobacteriaceae* are repeatedly reported as common members of not only chicken [9]–[11] but also human gut microbiota [21].

Representatives of *Desulfovibrionaceae* or *Campylobacteraceae,* which were detected among the core microbiota, are not frequently mentioned as common microbiota members. However, these may play important roles in the overall microbiota metabolism. Molecular hydrogen is released during fermentation by strict anaerobes of phyla *Firmicutes* and *Bacteroidetes* [22]. Since metabolism of such anaerobes is inhibited by the end product of their fermentation, *i.e.* by hydrogen, the hydrogen has to be removed. Both *Desulfovibrionaceae* and *Campylobacteraceae*, as well as *Megamonas* from the family *Veillonelaceae*, are capable of hydrogen utilization thus serving as a hydrogen sink in complex microbial communities [23], [24] which may explain the presence of *Desulfovibrionaceae*, *Campylobacteraceae* and *Megamonas* as common members of chicken microbiota. In addition, we observed a high correlation between the occurrence of *Bacteroidaceae* and *Desulfovibrionaceae* (Fig. 3). It has been described that the representatives of *Bacteroidaceae* are capable of sulphate release from sulphated chondroitin or mucin produced by host cells [25]. The released sulphate can in turn be respired to H_2_S by *Desulfovibrio* sp. [26] explaining the high correlation of *Desulfovibrionaceae* and *Bacteroidaceae*. Such ecological and metabolic bacterial mutualisms in complex microbiota systems may be used to prevent pathogen colonization. *E.g.* administering bacterial species from families *Desulfovibrionaceae* or *Veillonelaceae* with a similar metabolism and role as *Campylobacteraceae* to chickens may result in the replacement of *Campylobacteraceae* with *Desulfovibrionaceae* or *Veillonelaceae* and the reduction of *Campylobacter* sp. colonization in broilers, carcasses, and consequently poultry meat products.

## Material and Methods

### Ethics Statement

The owners of the farms were aware that the droppings were being collected for this study and gave their permission for doing so. Such permissions can be obtained from EPR for Croatian samples, IR for the Czech samples and SSM for Slovenian samples. Sampling of Hungarian broilers was performed on freshly slaughtered animals at two slaughter houses under the supervision and with the permission of the district veterinary officer and poultry owners. BN can be contacted for further information on Hungarian samples.

### Sample characterization

Altogether 137 fresh fecal samples originating from healthy flocks of both egg laying hens and broilers from 4 countries were analyzed in this study. Sixty-seven samples of fresh fecal droppings from 3 egg laying hen farms in the Czech Republic were taken, with 30, 28 and 9 samples collected at each farm. Fifteen fecal samples originated from 3 farms in Slovenia (five samples per farm). Ten of them originated from 3 and 4 week old broilers and the remaining 5 samples were collected from 62 week old laying hens. Thirty samples originated from 6 different farms in Croatia (five samples per farm). Twenty of these originated from 4 egg laying hen farms and the remaining 10 samples were collected at 2 different broiler farms. Thirty Hungarian samples originated from 3 different broiler farms, collecting 10 samples at each farm. Upon collection, the samples were kept frozen at −20°C preceding DNA purification. All the samples originated from farms following basically the same intensive production system of raising broilers or eggs with no antimicrobials having been provided while in egg or meat production.

### DNA purification and real-time PCR

After slowly defrosting at room temperature, approx. 250 mg of feces were homogenized for 1 min at 7000 RPM in a MagNALyzer (Roche Diagnostics) using zirconia silica beads (BioSpec Products). Following homogenization, the DNA was extracted using a QIAamp DNA Stool Mini Kit (Qiagen) according to the manufacturer's instructions and the purified DNA was stored at −20°C until use. The primer design for quantification of antibiotic resistance genes was described in our previous study [13] and all the primers are listed in Table 3. Target antibiotic resistance genes were selected to represent those commonly found in genomes of poultry microbiota [13], [27]. In addition, two primer pairs specific for the conservative regions of 16S rRNA genes (domain *Bacteria* universal primer pairs) were used to determine the total bacterial DNA present in the samples [28], [29]. Real-time PCR was carried out using QuantiTect SYBR Green PCR Kit (Qiagen) in a LightCycler 480 thermocycler (Roche). PCR was initiated with a hot start for 15 min at 95°C followed by 45 cycles of 20 sec at 95°C, 30 sec at 60°C and 30 sec at 72°C. Melting temperatures were determined after PCR to verify the correctness of each PCR product. The Ct values of the genes of interest were subtracted from an average Ct value of amplifications performed with *Bacteria* universal primer pairs (ΔCt) and the relative amount of each antibiotic resistance gene in the total bacterial population, *i.e.* antibiotic resistance gene prevalence in a given bacterial population, was finally calculated as 2^-ΔCt^.

**Table 3:** List of primers used in this study.

Primer	Target	Primer sequence 5′ - 3′	Reference
strA_F	aminoglycoside phosphotransferase	ACCCTAAAACTCTTCAATGC	[13]
strA_R	aminoglycoside phosphotransferase	TCCCCAATACATTGAATAGG	[13]
sul1_F	dihydropteroate synthase	GTCTAAGAGCGGCGCAATAC	[13]
sul1_R	dihydropteroate synthase	GGATCAGACGTCGTGGATGT	[13]
sul2_F	dihydropteroate synthase	CGCAATGTGATCCATGATGT	[13]
sul2_R	dihydropteroate synthase	GCGAAATCATCTGCCAAACT	[13]
tetB_F	tetracycline efflux protein	TACAGGGATTATTGGTGAGC	[13]
tetB_R	tetracycline efflux protein	ACATGAAGGTCATCGATAGC	[13]
tetA_F	tetracycline efflux protein	CGATCTTCCAAGCGTTTGTT	[13]
tetA_R	tetracycline efflux protein	CCAGAAGAACGAAGCCAGTC	[13]
cat_F	chloramphenicol acetyl transferase	GGGAAATAGGCCAGGTTTTC	[13]
cat_R	chloramphenicol acetyl transferase	TCCATGAGCAAACTGAAACG	[13]
16S_univ_F	all bacteria	GAGGAAGGIGIGGAIGACGT	[30]
16S_univ_R	all bacteria	AGICCCGIGAACGTATTCAC	[30]
16S_univ-1F	all bacteria	GTGSTGCAYGGYTGTCGTCA	[29]
16S_univ-1R	all bacteria	ACGTCRTCCMCACCTTCCTC	[29]

### Pyrosequencing

Forty-five samples representing 5 egg laying hen samples from each country and 10 broiler samples per country were selected for pyrosequencing and microbiota characterization. The purified DNA was used as a template in PCR with the forward primer 5′ CGTATCGCCTCCCTCGCGCCATCAG – MID-*GGAGGCAGCAGTRRGGAAT* 3′, and reverse primer 5′ CTATGCGCCTTGCCAGCCCGCTCAG- MID- *CTACCRGGGTATCTAATCC* 3′ using HotStarTaq Master Mix Kit following the manufacturer's instructions (Qiagen). The underlined sequences were required at different steps of pyrosequencing while those in italics are sequences complementary to the conserved parts of 16S rRNA genes flanking the V3/V4 hypervariable region [8], [30]. Cycling conditions consisted of a hot start at 95°C for 15 min followed by 30 cycles of incubation at 94°C for 40 s, 55°C for 55 s and 72°C for 60 s. PCR ended with a final extension at 72°C for 5 min. After PCR, the amplification products, approx. 525 bp in size, were separated electrophoretically in a 1.2% agarose gel, gel-purified using a QIAquick Gel Extraction Kit (Qiagen) and subjected to pyrosequencing. Pyrosequencing was performed using GS Junior Titanium sequencing chemistry and a GS Junior 454 sequencer according to the manufacturer's instructions (Roche).

### Sequence analysis

Fasta and qual files generated as an output of the pyrosequencing were uploaded into Qiime software [31]. Quality trimming criteria included no mismatch in MID sequences and a maximum of 1 mismatch in primer sequences. The obtained sequences with a qual score higher than 20 were shortened to the same length of 350 bp and classified with RDP Seqmatch with an OTU discrimination level set to 97%. In the next step, chimeric sequences were predicted and excluded from the analysis. Diversity analyses (rarefaction curves and Chao1 richness) on OTU clusters were performed using all sequences available for each sample. Finally, UniFrac analysis [32] followed by weighted principal coordinate analysis (PCoA) was used to characterize the diversity in the microbial populations tested. The raw sequence reads have been deposited in the NCBI Short Read Archive under the accession number SRP045877.

### Statistics

Data from real-time PCR are presented as medians ± 25^th^ and 75^th^ percentile. The comparison of antibiotic resistance gene representation in particular samples was evaluated by Kruskal-Wallis test using GraphPad Prism 5 software. A heat map based on Spearman's correlation coefficients was constructed in R using gplots package. Bipartite graphs of microbiota composition in different groups of birds were calculated using Matlab 2013a and visualized in Gephi 0.8.2.

## Supporting Information

Figure S1 **Composition of chicken fecal microbiota at the family level in 45 selected samples.**
(PDF)

Figure S2 **Comparison of broiler and egg layer fecal microbiota.**
(PDF)

Table S1 **List of raw Ct data for the quantification of antibiotic gene prevalence.**
(XLS)

Table S2 **Dominant phyla found in the samples analyzed in this study.**
(XLS)

Table S3 **List of all OTUs identified in this study.**
(XLS)
