# Supplementary material for: Characterization of Egg Laying Hen and Broiler Fecal Microbiota in Poultry Farms in Croatia, Czech Republic, Hungary and Slovenia
Source: PLoS One. 2014 Oct 16;9(10):e110076. doi: 10.1371/journal.pone.0110076 (PMC4199679; doi:10.1371/journal.pone.0110076)
Supplement: Figure S1 — Composition of chicken fecal microbiota at the family level in 45 selected samples. (PDF) [file pone.0110076.s001.pdf]

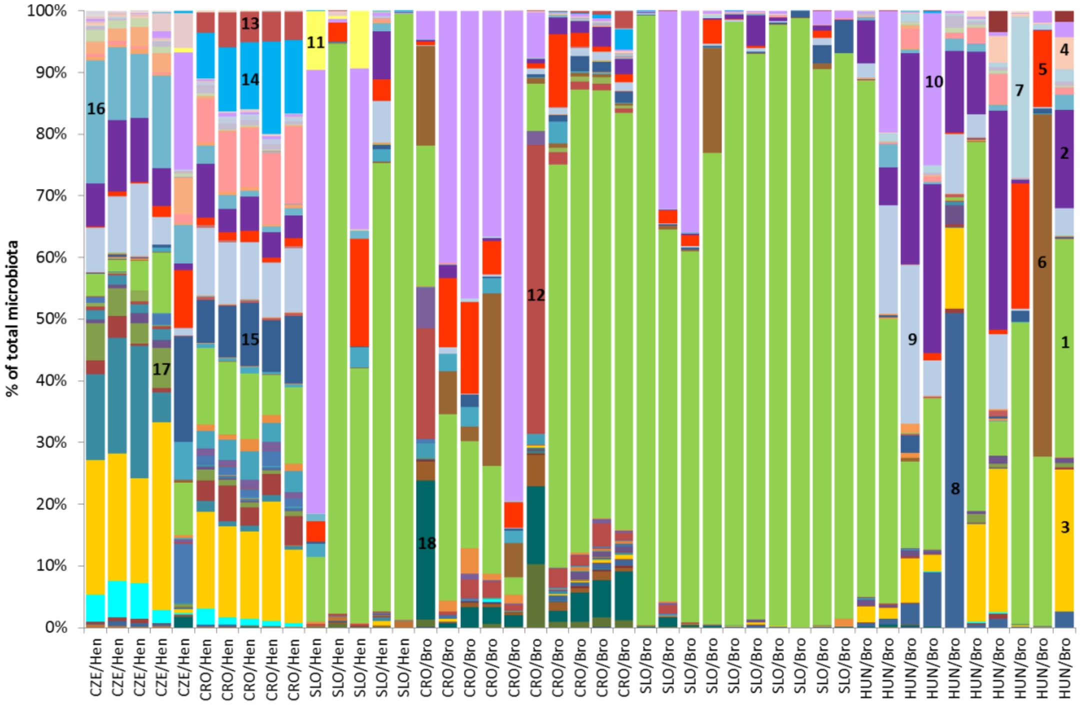

Figure S1. Composition of chicken fecal microbiota at the family level in 45 selected samples. Samples are arranged so that 5 samples in succession originate from the same farm. The most common bacterial families are indicated. 1 – *Lactobacillaceae*, 2 – *Ruminococcaceae*, 3 – *Bacteroidaceae*, 4 – *Helicobacteraceae*, 5 – *Peptostreptococcaceae*, 6 – *Streptococcaceae*, 7 – *Campylobacteraceae*, 8 – *Barnesiellaceae*, 9 – *Lachnospiraceae*, 10 – *Enterobacteriaceae*, 11 – *Pasteurellaceae*, 12 – *Staphylococcaea*, 13 – *Xanthomonadaceae*, 14 – *Pseudomonadaceae*, 15 – *Clostridiaceae*, 16 – *Veilonellaceae*, 17 – *Prevotellaceae*, 18 – *Corynebacteriaceae*, 19 – *Bacteroidaceae*. SLO – Slovenia, CRO- Croatia, CZE – Czech Republic, HUN – Hungary, Hen - samples from egg laying hens, Bro – samples from broilers.
