# Supplementary material for: Characterization of Egg Laying Hen and Broiler Fecal Microbiota in Poultry Farms in Croatia, Czech Republic, Hungary and Slovenia
Source: PLoS One. 2014 Oct 16;9(10):e110076. doi: 10.1371/journal.pone.0110076 (PMC4199679; doi:10.1371/journal.pone.0110076)
Supplement: Figure S2 — Comparison of broiler and egg layer fecal microbiota. (PDF) [file pone.0110076.s002.pdf]

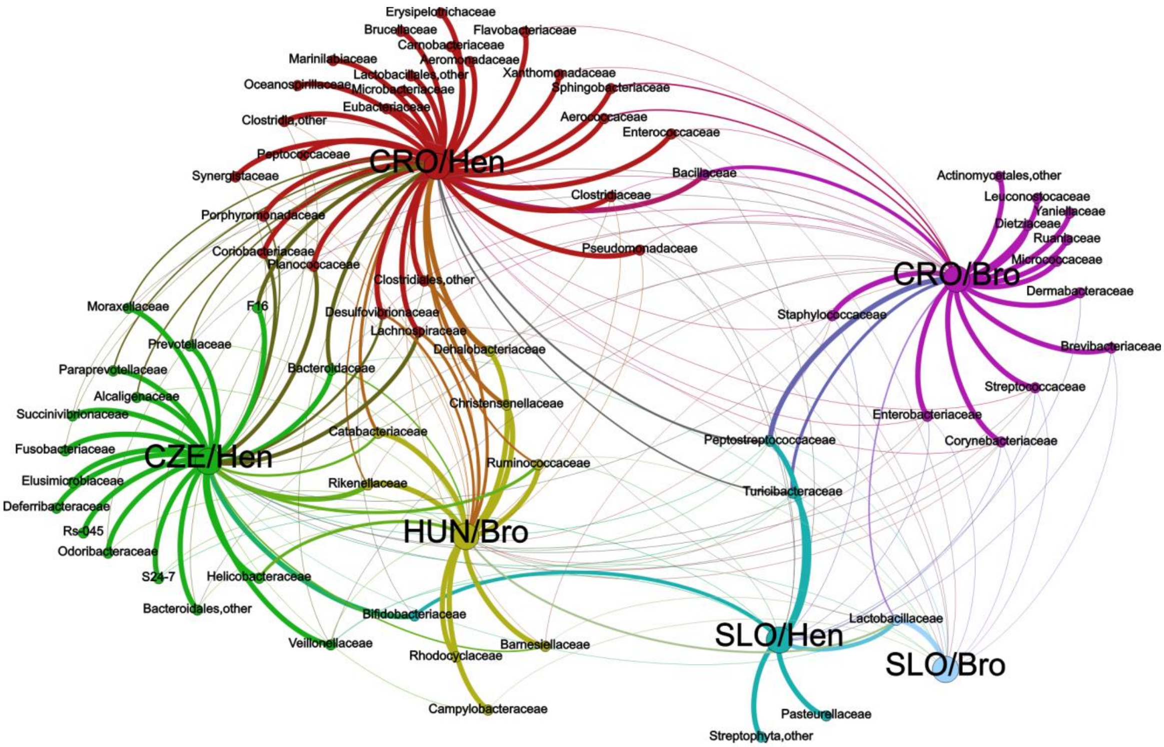

Figure S2. Microbiota composition in egg laying hens and broilers. Bipartite graph, edge width shows relative abundance of a family in different groups of laying hens or broilers. SLO – Slovenia, CRO- Croatia, CZE – Czech Republic, HUN – Hungary, Hen - samples from egg laying hens, Bro – samples from broilers.
